# *Csf1r*-mApple Transgene Expression and Ligand Binding In Vivo Reveal Dynamics of CSF1R Expression within the Mononuclear Phagocyte System

**DOI:** 10.4049/jimmunol.1701488

**Published:** 2018-02-12

**Authors:** Catherine A. Hawley, Rocio Rojo, Anna Raper, Kristin A. Sauter, Zofia M. Lisowski, Kathleen Grabert, Calum C. Bain, Gemma M. Davis, Pieter A. Louwe, Michael C. Ostrowski, David A. Hume, Clare Pridans, Stephen J. Jenkins

**Affiliations:** * Medical Research Council Centre for Inflammation Research, University of Edinburgh, Edinburgh EH16 4TJ, United Kingdom;; † The Roslin Institute, University of Edinburgh, Midlothian EH25 9RG, United Kingdom;; ‡ Faculty of Life Sciences, The University of Manchester, Manchester M13 9PL, United Kingdom;; § Hollings Cancer Center, Medical University of South Carolina, Charleston, SC 29425; and; ¶ Mater Research–University of Queensland, Translational Research Institute, Woolloongabba, Queensland 4104, Australia

## Abstract

CSF1 is the primary growth factor controlling macrophage numbers, but whether expression of the CSF1 receptor differs between discrete populations of mononuclear phagocytes remains unclear. We have generated a *Csf1r*-mApple transgenic fluorescent reporter mouse that, in combination with lineage tracing, Alexa Fluor 647–labeled CSF1-Fc and CSF1, and a modified Δ*Csf1–*enhanced cyan fluorescent protein (ECFP) transgene that lacks a 150 bp segment of the distal promoter, we have used to dissect the differentiation and CSF1 responsiveness of mononuclear phagocyte populations in situ. Consistent with previous *Csf1r-*driven reporter lines, *Csf1r*-mApple was expressed in blood monocytes and at higher levels in tissue macrophages, and was readily detectable in whole mounts or with multiphoton microscopy. In the liver and peritoneal cavity, uptake of labeled CSF1 largely reflected transgene expression, with greater receptor activity in mature macrophages than monocytes and tissue-specific expression in conventional dendritic cells. However, CSF1 uptake also differed between subsets of monocytes and discrete populations of tissue macrophages, which in macrophages correlated with their level of dependence on CSF1 receptor signaling for survival rather than degree of transgene expression. A double Δ*Csf1r*-ECFP-*Csf1r*-mApple transgenic mouse distinguished subpopulations of microglia in the brain, and permitted imaging of interstitial macrophages distinct from alveolar macrophages, and pulmonary monocytes and conventional dendritic cells. The *Csf1r-*mApple mice and fluorescently labeled CSF1 will be valuable resources for the study of macrophage and CSF1 biology, which are compatible with existing EGFP-based reporter lines.

## Introduction

The mononuclear phagocyte system (MPS) is a family of functionally related myeloid cells comprising progenitor cells, monocytes, macrophages, and conventional dendritic cells (cDC) (1–5). Macrophages resident in tissues may be derived from definitive hematopoiesis via circulating monocytes or by self-renewal from cells seeded during embryonic or early postnatal life (1, 2, 4, 5). cDCs have been classified as those cells deriving from common dendritic cell (DC) progenitors via circulating pre-DC (1, 6, 7). Regardless of their developmental origin, macrophages and their precursors express the M-CSF receptor CSF1R, and depend upon signals from two ligands, CSF1 or IL34, for proliferation, differentiation, and survival (2, 3). Receptor-mediated internalization and destruction of CSF1 controls its availability (8) and provides a homeostatic control on macrophage numbers (3). Accordingly, administration of recombinant CSF1 (9) or a more stable CSF1-Fc fusion protein (10–12) to mice produces expansion of blood monocyte and tissue macrophage populations, yet the degree to which CSF1R expression and activity differ between populations of mononuclear phagocytes is unclear.

cDC express high levels of a related tyrosine kinase receptor, Fms-like tyrosine kinase 3 (Flt3). Their numbers are greatly increased following treatment of mice with Flt3 ligand (Flt3L), and depleted in Flt3L-deficient animals (7, 13–17). Two subsets of cDC, cDC1 and cDC2, appear to differ in their expression of *Csf1r*. cDC1 are not generally dependent upon CSF1 and lack *Csf1r* mRNA (14). cDC2 have been more difficult to define because of major overlaps in cellular phenotype with other CD11b^+^CD11c^+^MHC class II (MHCII)^+^ monocyte-derived APCs (18–20). Genuine Flt3-dependent cDC2 of common DC progenitor origin have been defined based upon migratory behavior and the lack of the macrophage markers CD64 and F4/80 (6, 15, 21, 22). With this definition, cDC2 in various tissues expressed lower levels of *Csf1r* mRNA than monocyte-derived APC (15, 22) and have been considered CSF1R independent (1). The levels of surface CSF1R largely distinguish Flt3L-dependent cDC2 from short-lived monocyte-derived CD11c^+/−^ MHCII^+^ cells in the serous cavities (23, 24). However, cDC2 isolated from spleen express high levels of both *Csf1r* and *Flt3* mRNA (www.biogps.org) and their numbers are controlled by CSF1 in vivo (25). Therefore, it remains unclear whether there is a genuine dichotomy between Csf1r and Flt3-dependent myeloid APC.

CSF1R on macrophages is continuously removed from the cell surface by endocytosis and degraded following ligand binding. For that reason, the detection of CSF1R protein by immunohistochemistry or flow cytometry does not provide a clear indication of functional expression. To identify Csf1r-expressing cells in situ, regulatory elements of the murine *Csf1r* locus, including a 150 bp segment of the distal promoter, were used to produce *Csf1r-*EGFP reporter mice (26). The same promoter construct was used to drive constitutive (27) and inducible cre-recombinase to support macrophage-specific conditional mutations (28) as well as lineage tracing (29), and these tools have been widely distributed among the research community. However, new resources are required to verify with single-cell resolution the extent to which *Csf1r* transgene expression reflects that of functional CSF1R protein.

In addition to aiding our understanding of the regulation of myeloid cells, visualization of *Csf1r* gene and protein expression may also be useful to study cell interactions in vivo due to the lack of tools to identify discrete MPS populations during multicolor imaging. A binary enhanced cyan fluorescent protein (ECFP) reporter (Δ*Csf1r*-Gal4VP16/UAS-ECFP) transgene with a 150 bp segment of the distal Csf1r promoter deleted, termed Δ*Csf1r*-ECFP, has provided a novel tool to support in vivo imaging of monocyte trafficking (30, 31), because expression was lost from the large majority of tissue macrophages but remained in blood monocytes, microglia, Langerhans cells, and cDC2 (32). In particular, dual reporter mice, such as those generated by crossing the *Cx3cr1*-EGFP and *Ccr2*-RFP mice, have been valuable tools for visualizing monocyte subsets and their differentiation in the brain and liver (33), findings that would not have been obtainable using single reporter mice. However, many other macrophage and nonmacrophage reporter genes use EGFP, rendering the original *Csf1r*-EGFP transgene of limited use for this purpose. Thus, additional monocyte/macrophage reporter mice that are compatible with existing EGFP-based reporters are needed.

Hence, we have created new tools and assays to image and assess *Csf1r* gene and protein expression that can be combined conveniently with common fluorophores, EGFP transgenes, and the Δ*Csf1r*-ECFP transgene for use in imaging and flow cytometry. In particular, we characterize a new *Csf1r*-mApple line expressing the red reporter gene *mApple* under the same promoter used in the *Csf1r*-EGFP reporter, and apply this in combination with the Δ*Csf1r*-ECFP transgene, lineage tracing, and labeled CSF1-Fc and CSF1 proteins to distinguish different cellular compartments within the MPS, and to dissect the homeostatic roles of CSF1.

## Materials and Methods

### Plasmid constructs

The 7.2 kb *Csf1r* reporter construct previously used to generate the *Csf1r-*EGFP mice (26) was digested with ApaI and SalI (NEB) to remove EGFP before gel purification using the QIAquick gel extraction kit (Qiagen). Overhangs were removed with Mungbean nuclease (NEB) and DNA was purified using QIAGEN MinElute columns (Qiagen), then dephosphorylated using thermosensitive alkaline phosphatase (Promega). A construct encoding the fluorescent protein *Csf1r*-mApple (34) was digested with SmaI and AflII, similarly purified, and overhangs removed before both constructs were precipitated with EtOH/NaOAc and then ligated with T4 ligase (NEB) at 16°C overnight. The resulting *Csf1r*-mApple construct was transformed into DH5α competent cells. The *Csf1r-rtTA-M2* construct utilizing the same 7.2 kb mouse *Csf1r* promoter region was used previously to generate *Csf1r-rtTA* transgenic mice (35) For generation of transgenic mice, plasmid backbones were removed by digestion with DrdI/PvuI (*Csf1r*-mApple, NEB) and SalI/MluI (*Csf1r-*rtTA, Promega/NEB) and then gel-purified using a QIAquick gel extraction kit. DNA was then further purified using AMPure XP beads (Agencourt) according to the instructions.

### Generation of transgenic mice and animal maintenance

Animal experiments were permitted under license by the U.K. Home Office, and were approved by the University of Edinburgh Animal Welfare and Ethical Review Body. All mice including wild-type (WT) C57BL/6JOlaHsd CD45.2^+^, congenic CD45.1^+^CD45.2^+^, *Csf1r*-EGFP (26), Δ*Csf1r*-Gal4VP16/UAS-ECFP (36), and Ccr2^−/−^ (37) lines were bred and housed in specific-pathogen free facilities at the University of Edinburgh. *Csf1r-mApple/Csf1r-rtTA* transgenic mice were generated at the University of Edinburgh’s Central Biological Services Transgenic Core facility by microinjection of transgenes into the pronuclei of fertilized oocytes from C57BL/JOlaHsd mice. The integration of the transgenes was determined by PCR analysis of genomic DNA isolated from ear biopsy using primers that amplified a 565 bp product between the c-fms promoter and rtTA gene, and a 507 bp product between the c-fms promoter and *Csf1r-mApple* gene, using primers 5′-TTC CAG AAC CAG AGC CAG AG-3′ (forward) and 5′-CTG TTC CTC CAA TAC GCA GC-3′ (reverse), and 5′-CCT ACA TGT GTG GCT AAG GA-3′ (forward) and 5′-CTT GAA GTA GTC GGG GAT GT-3′ (reverse), respectively, and amplification temperatures of 35 cycles of 30 s at 94, 55, and 72°C, after an initial denaturing step of 94°C for 5 min. Expression of *Csf1r-mApple* was verified by screening 10 μl blood for the presence of *Csf1r*-mApple fluorescence by flow cytometry. One founder positive for both transgenes transmitted the transgenes to progeny and established the *Csf1r*-mApple*/Csf1r-rtTA* line (referred to as *Csf1r*-mApple). The *Csf1r*-mApple line was maintained by breeding to C57BL/6JOlaHsd mice, or where specified, bred to the Δ*Csf1r*-ECFP line, for which subsequent analysis was performed on F1 progeny. For maintenance of the *Csf1r*-mApple line, transgenic progeny were initially identified by PCR analysis of genomic DNA and flow-cytometric assessment of the presence of *Csf1r-*mApple in blood cells, and subsequently by flow cytometry alone. For identification of myeloid populations replenished by CCR2-dependent bone marrow (BM) precursors, tissue-protected BM chimeric mice were generated as previously described (23). Briefly, anesthetized C57BL/6 CD45.1^+^CD45.2^+^ congenic mice were exposed to a single dose of 9.5 Gy γ-irradiation, while all but the hind legs and lower abdomen were protected by a 2 inch lead shield. Animals were subsequently given 2–5 × 10^6^ BM cells from CD45.2^+^ C57BL/6J mice or *Ccr2*^−/−^ animals by i.v. injection before being left for 8 wk prior to analysis of chimerism in the tissue compartments. All experiments were performed with age- and sex-matched littermate control mice and approved by the University of Edinburgh Animal Welfare and Ethical Review Body under license granted by the U.K. Home Office.

### Tissue digestion and FACS analysis

Unless otherwise stated, mice were culled by a rising concentration of CO_2_. Then 100 μl of blood was collected by cardiac puncture into EDTA tubes. The peritoneal cavity was lavaged with RPMI 1640 containing 2 mM EDTA and 10 mM HEPES (Invitrogen). Cadavers were subsequently perfused and lung and liver removed and chopped finely, and digested in prewarmed collagenase mixture [0.625 mg ml^−1^ collagenase D (Roche), 0.85 mg ml^−1^ collagenase V (Sigma-Aldrich), 1 mg ml^−1^ dispase (Life Technologies, Invitrogen), and 30 U ml^−1^ DNase (Roche Diagnostics) in RPMI 1640] for 22 and 45 min respectively in a shaking incubator at 37°C before being passed through a 100 μm filter. Lung preparations were washed in PBS containing 2 mM EDTA (Life Technologies, Invitrogen) and 0.5% BSA (Sigma-Aldrich), termed FACS buffer, followed by centrifugation at 300 g for 5 min, whereas liver preparations were washed in 50 ml then 30 ml of ice-cold RPMI 1640 followed by centrifugation at 300 g for 5 min. Erythrocytes in tissues and blood were lysed using RBC lysis buffer from Sigma-Aldrich or BioLegend, respectively. All cells were maintained on ice until further use. Cellular content of the preparations was assessed by cell counting using a CASY TT counter (Roche). Equal numbers of cells or equivalent volumes of blood were stained with Zombie Aqua viability dye (Invitrogen) blocked with 0.025 μg anti-CD16/32 (2.4G2; BioLegend) and 1:10 heat-inactivated mouse serum (Invitrogen), and then surface stained with a combination of Abs in FACS buffer. The following Abs were used: F4/80 (BM8), Siglec-F (E50-2440), Siglec-F (ES22-10D8), Ly6C (HK1.4), CD11b (M1/70), CD11c (N418), MHCII (M5/114.15.2), CD19 (6D5), CD3 (17A2), CD3 (17A2), CSF1R (AFS98), CD45.1 (A20), CD45.2 (104), CD226 (10E5), CD64 (X57-5/7), Ly6G (1A8), CD26 (H194-112), and PDCA-1 (927) (eBioscience, Miltenyi Biotec, or BD Europe). Where applicable, cells were subsequently stained with streptavidin-conjugated fluorochromes. Fluorescence minus one controls confirmed gating strategies, whereas discrete populations within lineage-negative cells were confirmed by omission of the corresponding population-specific Ab. Samples were acquired on an LSRFortessa flow cytometer (Becton Dickinson) at the Queens Medical Research Institute Flow Cytometry and Cell Sorting Facility and resulting data were analyzed using FlowJo V9 software. CD45^+^ cells were identified as live single cells by excluding 7AAD^+^ or Zombie Aqua^+^ cells and using forward scatter area versus forward scatter height characteristics. Cells positive for CD19, CD3, Ly6G, and SiglecF, or CD19, CD3, and Ly6G were referred to as Lineage^+^ and were excluded prior to analysis of liver, blood and cavity cells, or lung cells, respectively, as shown in the respective figures.

For the processing of brain tissue, double transgenic mice were perfused transcardially with physiological saline and brains were removed for regional dissection into cerebellum, cortex, hippocampus, and striatum. Mixed brain cell homogenates were prepared as described (32). The single-cell suspension of each region was incubated with 1 μg ml^−1^ anti-CD16/32 and subsequently stained with rat anti-mouse/human CD11b (M1/70) and rat anti-mouse CD45 (BioLegend). Flow cytometry was acquired using the Fortessa ×20 (Becton Dickinson) and resulting data were analyzed using FlowJo V10 software.

### Inhibition of CSF1R signaling

The CSF1R kinase inhibitor GW2580 (LC Laboratories) was suspended in 0.5% hydroxypropylmethylcellulose and 0.1% Tween 20 using a Teflon glass homogenizer. Diluent control or 160 mg/kg GW2580 was administered daily for 4 d by oral gavage before mice were culled on day 5.

### Alexa Fluor 647–labeled CSF1 and anti-CSF1R mAb

Preservative-free sterile anti-CSF1R mAb (clone AFS98) was purchased from Bioserv (Sheffield, U.K.). Porcine CSF1 and CSF1-Fc was prepared as described previously (12). CSF1 and CSF1-Fc were conjugated to Alexa Fluor 647 (AF647) using the AF647 Microscale Protein labeling kit from Thermo Fisher Scientific according to manufacturer’s instructions, and sodium azide subsequently removed using 7k MWCO Pierce polyacrylamide spin desalting columns (Thermo Fisher Scientific). Mice were injected i.v. with 0.5 mg anti-CSF1R mAb or PBS vehicle control, followed by 5 μg CSF1-Fc^AF647^ or PBS vehicle i.v. 10 min later. After a further 10 min, 60 μl of blood was removed by tail venipuncture, with the animals then immediately culled by cervical dislocation, and tissues perfused with PBS through the inferior vena cava. For study of CSF1 uptake in the peritoneal cavity, mice were injected i.p. with or without 0.5 mg AFS98 followed by 0.5 μg CSF1^AF647^ or PBS vehicle 2 min later, and then culled 10 min later by exposure to increasing levels of CO_2_. The degree of CSF1R-dependent uptake of CSF1^AF647^ or CSF1-Fc^AF647^ is presented as the Δ median fluorescence intensity (MFI) calculated as the MFI for individual samples from mice given labeled CSF1 minus the average MFI from all samples pretreated with AFS98.

### Imaging of tissues and cells

Whole-mount imaging of freshly isolated tissues from transgenic and WT littermate control mice aged 12–15 wk was performed using a Zeiss AxioZoom.V16 fluorescence microscope. Immediately after excision, tissues were kept at 4°C and protected from light. The fluorescent signal was acquired at 500–550 and 590–650 nm for EGFP and *Csf1r*-mApple, respectively. Acquisition of tissue background signal was performed by imaging WT tissue with the filter used for detection of the *Csf1r*-mApple protein.

### Ex vivo confocal imaging of tissues

Male transgenic or WT male littermates were anesthetized, as per regulations, and intravenously injected in the tail vein with 5 μg/g of weight of Lectin-I [from *Griffonia* (*Bandeiraea*) *simplicifolia*] tagged with FITC (Vector Labs). After 10 min, mice were perfused transcardially with HBSS (Thermo Fisher Scientific), at a rate of 10 ml per min, and the left lobe of the liver was excised. Lungs were inflated with a solution containing 1% low melting-point agarose (Sigma-Aldrich) and, upon agarose solidification, the left lung was excised. Detection of functional CSF1R in lung myeloid subsets was performed by administering 5 μg CSF1-Fc^AF647^ and 5 μg/g of weight of Lectin-I via i.v. injection. Mice were perfused with HBSS, previous to lung excision and inflation with agarose, as described. After dissection, liver and lung were placed on coverslip-bottom chambers and covered with a sufficient volume of HBSS to prevent the surface of tissues from drying. Chambers were kept on ice and protected from light until tissues were imaged on a Zeiss LSM 710 microscope. Laser wavelengths for ECFP, FITC, and mApple were 405, 488, and 543 nm, respectively. Fluorescence acquisition for ECFP, FITC, and mApple signals in liver and lung was 400–480, 525–600, and 602–758 nm, respectively. Acquisition settings for lung tissue treated with CSF1-Fc^AF647^ were 400–479, 525–583, 593–651, and 651–755 nm for ECFP, FITC, mApple, and AF647, respectively. Postprocessing of images was performed by adjusting the black/white thresholds in the software ZEN 2012 (blue edition) developed by Carl Zeiss as follows: ECFP: 0–175, FITC: 0–100, mApple: 0–75, AF647: 0–200.

### Statistics

Statistical tests detailed in the figure legends were performed using GraphPad Prism 6. Where necessary, data were log-transformed to achieve equal variance.

## Results

### Generation of Csf1r-mApple mice

C57BL/6 mouse embryos were comicro-injected with a construct containing the 7.2 kb *Csf1r* promoter region used to create the *Csf1r*-EGFP mice (26) upstream of *mApple*, along with a construct encoding the reverse tetracycline inducible transactivator *rtTA-m2* under control of the same promoter (*Csf1r-*rtTA), previously used to generate a *Csf1r*-driven Tet-on system (35). mApple was used because it is brighter than its parent mCherry, refractory to photobleaching (38), suffers little from background autofluorescence, and previously enabled whole-mount imaging of the avian response to CSF1 in *Csf1r*-mApple reporter chickens (39). A single founder positive by PCR for both transgenes and for mApple protein in blood cells by flow cytometry was mated with a WT C57BL/6 mouse to establish the *Csf1r*-mApple line. PCR analysis across 77 mice revealed that *Csf1r-*mApple and *Csf1r-*rtTA transgenes were exclusively coinherited, suggesting cointegration (data not shown). PCR and flow cytometry analysis of blood demonstrated the *Csf1r-*mApple transgene to be inherited at a frequency of 44.0% (*n* = 207). The utility of the cointegrated Tet-on cassette is under investigation and is not considered further in this study but preliminary data demonstrate *rtTA-m2* mRNA is expressed in peritoneal cells (data not shown).

### Comparison of Csf1r-EGFP and Csf1r-mApple expression across tissue

**FIGURE 1.**
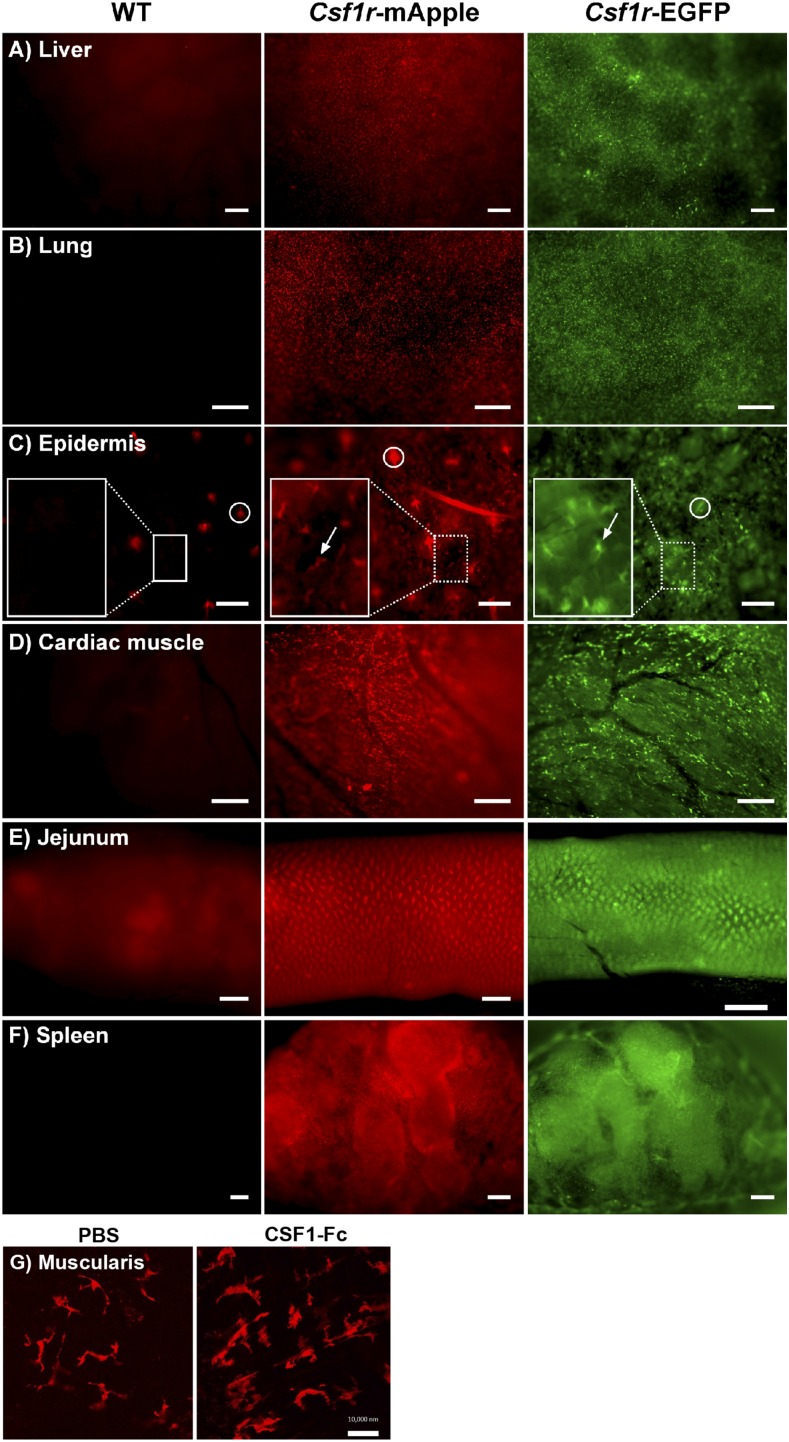
Distribution of the *Csf1r*-mApple and *Csf1r*-EGFP transgenes is similar throughout tissues. Whole-mount imaging of freshly isolated liver (**A**), lung (**B**), skin epidermis (**C**), cardiac muscle (**D**), jejunum (**E**), and cross-section of spleen (**F**) from WT (left panel), *Csf1r*-mApple (central), and *Csf1r*-EGFP (right panel) mice, or from muscularis (**G**) of *Csf1r*-mApple mice treated with PBS (left panel) or CSF1-Fc (right panel). In (C), a region of interest has been further magnified to better show Langerhans cells (white arrows), and hair follicles can be detected as highly autofluorescent structures (white circles). Scale bars in skin represent 100 μm; 200 μm in liver, spleen, and cardiac muscle; 500 μm in lung and jejunum; and 10 μm in the muscularis.

In whole-mount fluorescence microscopy of live organs from *Csf1r-*mApple mice expression patterns of mApple recapitulated EGFP in *Csf1r*-EGFP transgenic mice (Fig. 1A–F). Large stellate mApple^+^ cells were observed throughout the liver, lung, epidermis, and cardiac muscle. Both transgenic strains highlighted the abundant macrophage populations of the intestinal lamina propria (Fig. 1E), and the red pulp of spleen (Fig. 1F) (26). Background fluorescence in littermate control mice was negligible (Fig. 1A–F, left panel). The *Csf1r*-EGFP and Δ*Csf1r*-ECFP transgenes have been used extensively for in vivo imaging with multiphoton and spinning disc microscopes (e.g., Refs. 20, 30, 31, 40–43), providing high-resolution analysis of macrophage motility and the extent of their ramified processes. Multiphoton imaging of whole mounts of the muscularis externa of the intestine demonstrated the high signal-to-noise ratio obtainable with the *Csf1r*-mApple reporter (Fig. 1G), enabling visualization of the regular network of microglial-like macrophages in this site (44, 45). Furthermore, the impact of exogenous CSF1-Fc, which regulates the function of these cells (44), could be directly visualized as an increase in cell size.

### Csf1r-mApple expression by blood myeloid cells

**FIGURE 2.**
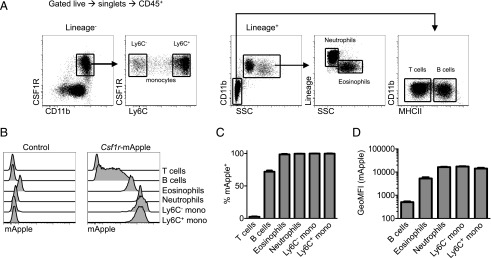
*Csf1r*-mApple transgene expression in blood leukocytes. (**A**) Flow cytometric strategy to identify blood leukocytes. (**B**) Expression of *Csf1r*-mApple in venous blood leukocytes from a representative *Csf1r-*mApple (right) and WT littermate control (left) mouse. (**C**) Frequency of cells positive for *Csf1r-*mApple and (**D**) geometric mean fluorescence intensity (GeoMFI) of *Csf1r-*mApple gated on *Csf1r-*mApple^+^ cells for different blood leukocytes. (B–D) Representative data from one of three experiments. Data are presented as mean ± SD of four mice (C and D).

To determine efficiency, reliability, and specificity of transgene expression, flow cytometry was performed on the blood of a cohort of *Csf1r*-mApple mice and littermate controls. Circulating CSF1R^+^CD11b^+^ monocytes (Fig. 2A) were uniformly *Csf1r*-mApple^+^ in both Ly6C^+^ and Ly6C^−^ subsets (Fig. 2B, 2C). As reported for the expression of EGFP in *Csf1r*-EGFP mice, neutrophils and eosinophils, which express *Csf1r* mRNA but not protein (46), were also *Csf1r-*mApple positive (Fig. 2B, 2C). The *Csf1r* promoter is active in B cells, which like macrophages, express the key transcription factor, PU.1, albeit at lower levels (47). Accordingly, ∼70% of B cells had very low, but detectable, *Csf1r-*mApple (Fig. 2B, 2C). Similar expression of EGFP in *Csf1r*-EGFP mice is not detectable by confocal microscopy on spleen sections (46). The intensity of *Csf1r*-mApple expression was consistent between animals, with equivalent levels expressed by monocytes and neutrophils and lower levels in eosinophils and B cells (Fig. 2D). Hence, the pattern of *Csf1r*-mApple expression reproduced that reported for EGFP in *Csf1r*-EGFP mice.

### The level of Csf1r-mApple expression distinguishes monocytes, macrophages, and cDC in different tissues

**FIGURE 3.**
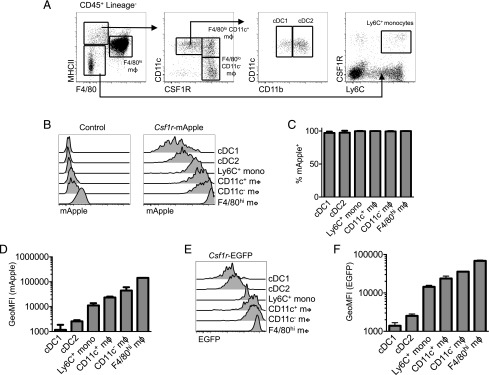
*Csf1r* transgene and CSF1R protein expression in the peritoneal cavity. (**A**) Flow cytometric strategy to identify peritoneal cavity myeloid cells as recently described (23). (**B**–**D**) Expression of *Csf1r-*mApple in peritoneal cavity myeloid populations determined by flow cytometry, showing a representative WT littermate control (left) and *Csf1r*-mApple (right) mouse (B), and graphs depicting the mean frequency of *Csf1r-*mApple^+^ cells in each population (C) and geometric mean fluorescence intensity (GeoMFI) of *Csf1r-*mApple for different peritoneal leukocytes gated on *Csf1r*-mApple^+^ cells (D). (**E** and **F**) Expression of *Csf1r*-EGFP in peritoneal cavity myeloid populations from *Csf1r*-EGFP mice, showing a representative flow cytometric overlay (E) and graphs depicting the frequency and GeoMFI of *Csf1r*-EGFP^+^ cells in each population (F) across multiple mice. Representative data from one of three experiments (B–D) or a single experiment (E and F). Data are presented as mean ± SD of four mice (C, D, and F).

To determine if transgene expression distinguished cDC and macrophages across multiple tissues, we first confirmed the identity of marker-defined MPS populations before surveying transgene expression in *Csf1r-*mApple mice. In the peritoneal cavity, we have demonstrated that recruited monocytes continuously replenish rare short-lived F4/80^lo^ MHCII^+^ macrophages that include both CD11c^+^ and CD11c^−^ cells (23), although only slowly replacing the more abundant F4/80^hi^ resident macrophages of embryonic origin (23). Both CD11c^+^ and CD11c^−^ short-lived and F4/80^hi^ peritoneal macrophage populations express detectable surface CSF1R. In contrast, Flt3-dependent cavity cDC of nonmonocyte BM origin also express CD11c^+^ and MHCII^+^ and can be found among F4/80^lo/−^ cells, but can be distinguished as CSF1R^−^ (23, 24, 48, 49). Based upon this published gating strategy and previously assigned ontogenies (Fig. 3A) (23), *Csf1r-*mApple was detected in Ly6C^+^ monocytes, all macrophage populations, and in CD11b^−^ cDC1 and CD11b^+^ cDC2 (Fig. 3B, 3C) (24). There was a progressive increase in *Csf1r-*mApple intensity between Ly6C^+^ monocytes, CD11c-defined subsets of short-lived F4/80^lo^MHCII^+^ macrophages, and long-lived F4/80^hi^ macrophages (Fig. 3D), consistent with the linear developmental relationship between these populations and monocytes in adult mice (23, 48, 50). *Csf1r*-mApple fluorescence in both CD11b^−^ cDC1 and CD11b^+^ cDC2 was lower than in monocytes (Fig. 3D), consistent with the lack of surface CSF1R (Fig. 3A). EGFP expression in *Csf1r*-EGFP mice replicated this pattern (Fig. 3E, 3F).

**FIGURE 4.**
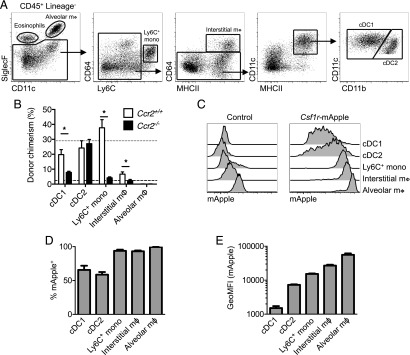
Lineage-restricted *Csf1r*-mApple transgene expression in the lung. (**A**) Flow cytometric strategy to identify lung leukocytes. (**B**) Frequency of donor cells within each lung population from tissue protected BM chimeric mice 8 wk after receiving BM from WT (white) or *Ccr2*^−/−^ (black) mice. Mean donor chimerism for circulating Ly6C^+^ monocytes is presented as short- or long-dashed lines for recipients of WT or *Ccr2*^−/−^ BM, respectively. (**C**) Expression of *Csf1r*-mApple in lung leukocytes from a representative WT littermate control (left) and *Csf1r*-mApple (right) mouse. (**D**) Frequency of cells expressing *Csf1r-*mApple and (**E**) geometric mean fluorescence intensity (GeoMFI) of *Csf1r*-mApple gated on *Csf1r-*mApple^+^ cells for different lung leukocytes. Data are from one of two independent experiments. Data presented as mean ± SD of four mice (D and E) or mean ± SEM of five mice (B). The asterisk (*) indicates significant differences using multiple *t* tests corrected for multiple comparisons using the Holm–Sidak method.

In the lung, alveolar macrophages are readily identified based upon high levels of CD11c and SiglecF (Fig. 4A) (51, 52). Interstitial cells are more heterogeneous. Some MHCII^+^ cells with varying levels of CD11c have been defined as macrophages based upon their expression of the Fc receptor CD64 and CSF1R dependence (51, 53), which contrasts the Flt3 dependence of CD64^−^ interstitial cDC2 (15). To verify that CD64 expression distinguishes pulmonary interstitial macrophages from CD11b^+^ cDC2, we assessed the turnover kinetics of CD64-defined MHCII^+^ cells and their dependence on CCR2, an established method for determining the likely monocyte dependence of tissue MPS cells (23, 54). We used a BM chimeric system in which WT mice were irradiated with organs of interest shielded to prevent irradiation-induced injury and reconstituted with congenic WT or *Ccr2*^−/−^ BM. This approach results in stable nonhost chimerism in blood leukocytes of ∼30% in recipients of WT BM (23, 55) (Fig. 4B, short-dashed line) and allows the turnover kinetics of tissue populations to be assessed. Importantly, in recipients of *Ccr2*^−/−^ BM chimerism in monocytes (Fig. 4B, long-dashed line) but not other circulating leukocytes is largely abolished (23). Notably, putative CD64^−^CD11b^+^MHCII^+^ cDC2 were completely replaced within 8 wk, consistent with the short half-life of DCs (14). This occurred in a completely CCR2-independent manner, with identical chimerism in recipients of WT and *Ccr2*^−/−^ BM (Fig. 4B). In contrast, relatively few CD64^+^MHCII^+^ cells were replaced over 8 wk, although this was completely dependent on CCR2, suggesting slow replenishment from monocytes. Thus, consistent with previous work (15), CD64 accurately defines distinct CD11b^+^ MPS populations. Alveolar macrophages showed no evidence of chimerism (Fig. 4B), consistent with self-maintenance (52, 56, 57). Surprisingly, replenishment of cells defined as cDC1 was also dependent upon CCR2. However, these cells express CCR2 within the lung environment (58) and thus may require this receptor for tissue retention. Based upon the verified ontogenies, Ly6C^+^ monocytes, CD64^+^ interstitial macrophages, and alveolar macrophages in the lungs were all *Csf1r*-mApple^+^ (Fig. 4C, 4D), but expression increased progressively between monocytes and mature macrophages (Fig. 4C, 4E). Both cDC populations also expressed *Csf1r*-mApple, but at lower levels than monocytes (Fig. 4C–E).

**FIGURE 5.**
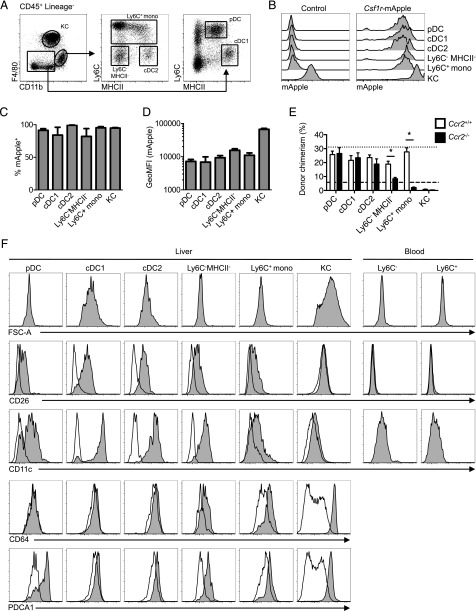
*Csf1r*-mApple transgene expression in the liver. (**A**) Flow cytometric strategy to identify liver leukocytes. (**B**) Expression of *Csf1r*-mApple in liver leukocytes with histograms from a representative *Csf1r*-mApple (right) and WT littermate control (left) mouse. (**C**) Frequency of cells expressing *Csf1r*-mApple and (**D**) geometric mean fluorescence intensity (GeoMFI) of *Csf1r*-mApple gated on *Csf1r*-mApple^+^ cells for different liver leukocytes. (**E**) Frequency of donor cells within each hepatic population from tissue protected BM chimeric mice 8 wk after receiving BM from WT (white) or *Ccr2*^−/−^ (black) mice. Mean donor chimerism for blood Ly6C^+^ monocytes is presented as short- or long-dashed lines for recipients of WT or *Ccr2*^−/−^ BM, respectively. (**F**) Representative histograms showing forward scatter area (FSC-A) characteristics and CD26, CD11c, CD64, and PDCA1 expression (tinted) overlayed with FMO controls (open) for liver leukocytes and blood monocytes. (B–D) Representative data from one of three or (E) two experiments, with data presented as mean ± SD of four mice (C and D) or mean ± SEM of five (E) mice. The asterisk (*) indicates significant differences using multiple *t* tests corrected for multiple comparisons using the Holm–Sidak method.

In the liver, the largest phagocyte population is the Kupffer cells (KC), but a minority CD11b^+^F4/80^lo^ BM-derived population may include monocytes, cDC2, and possibly F4/80^lo^ BM-derived macrophages (14, 29, 57). KCs [F4/80^hi^CD11b^lo^ (29, 59, 60)] (Fig. 5A) exhibited uniformly high expression of *Csf1r*-mApple (Fig. 5B–D). The minority CD11b^+^F4/80^lo^ compartment was subdivided based upon Ly6C and MHCII (Fig. 5A). The Ly6C^+^ cells and Ly6C^−^MHCII^−^ cells resembled Ly6C^+^ and Ly6C^−^ blood monocytes in size and marker expression (Fig. 5F). Unlike blood monocytes, MHCII^+^ cells among CD11b^+^F4/80^lo^ cells were larger, exhibited high levels of CD11c, and must include the Flt3-dependent CSF1R-independent CD11b^+^ cDC2 described previously (14); hence, we provisionally assigned these cDC2 (Fig. 5F). All CD11b^+^ populations expressed high levels of *Csf1r-*mApple (Fig. 5B, 5C). In the same CCR2-dependent tissue-protected BM chimera system used for the lung, the putative CD11b^+^ cDC2 population was replenished almost entirely by CCR2-independent BM precursors (Fig. 5E). They also expressed the highest levels of CD26, a marker of cDC conserved across species (61), and were negative for CD64 (Fig. 5F), confirming them as cDC2. CD11b^−/lo^F4/80^−^ cells were also positive for *Csf1r*-mApple, but expressed markers of cDC1 (Ly6C^−^MHCII^+^CD11c^+^CD26^+^) and plasmacytoid DC (pDC) (Ly6C^+^MHCII^+^PDCA-1^+^) (Fig. 5A, 5F), and were replenished by CCR2-independent precursors (Fig. 5E). Hence, in liver the *Csf1r* transgene did not distinguish cDC from monocytes, but was highest in mature macrophages.

### Detection of functional CSF1R using fluorescent CSF1-Fc

**FIGURE 6.**
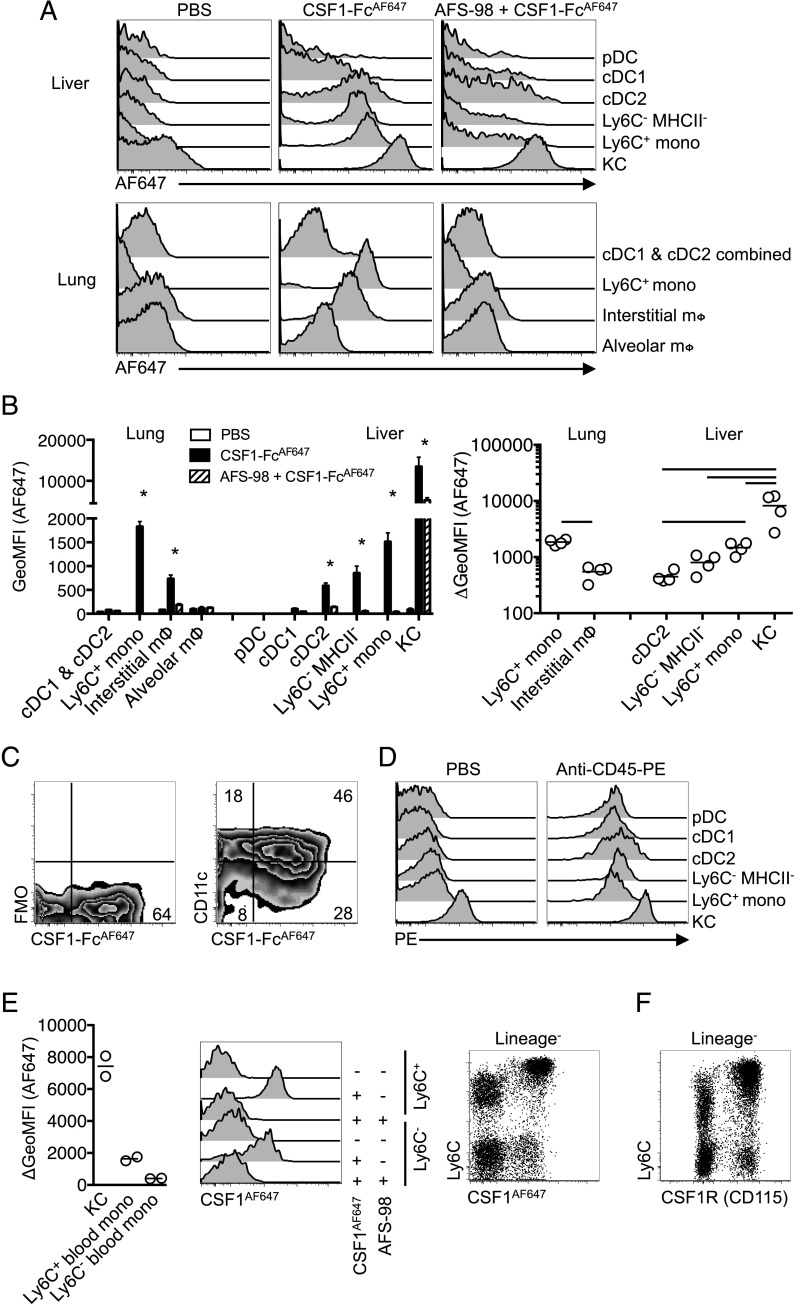
Tissue-specific consumption of CSF1 by cDC2 in vivo. (**A**) Histograms of AF647 fluorescence of leukocyte populations from lung and liver following i.v. injection of anti-CSF1R mAb AF-S98 or PBS vehicle before subsequent i.v. delivery of CSF1-Fc^AF647^ or PBS vehicle. (**B**) Geometric mean fluorescence intensity (GeoMFI) of AF647 of cells from mice in (A) (left graph) and change in MFI between mice given CSF1-Fc^AF647^ alone or after AFS-98 pretreatment (right graph), with data presented as mean ± SEM of four mice per group (left), or with data points for individual mice shown (right). Data are from one of two repeat independent experiments. (**C**) Representative plots showing CSF1-Fc^AF647^ uptake versus CD11c expression or FMO control on hepatic cDC2 from mice in (A). (**D**) PE fluorescence of liver leukocytes from C57BL/6 mice injected i.v. with anti–CD45-PE mAb or PBS vehicle 2 min prior to necropsy, showing data from one representative mouse of two per group. (**E**) Histograms of AF647 fluorescence of Ly6C^+^ and Ly6C^−^ blood monocytes from mice treated as in (A) but given CSF1^AF647^, and a graph showing change in GeoMFI of blood monocytes and liver KC between mice given CSF1^AF647^ alone or after pretreatment with AFS98, and a dot plot showing Ly6C versus AF647 uptake on all CD3^−^CD19^−^Ly6G^−^ blood cells with data points representing individual mice. (**F**) Conventional surface CSF1R (CD115) and Ly6C staining on CD3^−^CD19^−^Ly6G^−^ blood cells from naive mice. Data are from one representative experiment of two (B–D and F) or three (E) independent repeats. The asterisk (*) indicates significant differences between CSF1-Fc^AF647^ alone and AFS98 + CSF1-Fc^AF647^ using *t* tests corrected for multiple comparisons with the Holm–Sidak method (B, left graph), whereas lines indicate significant differences using one-way ANOVA (B, right graph).

*Csf1r* mRNA may be posttranscriptionally regulated (62) and the protein may be cleaved from the cell surface in response to TLR signals (63). To assess functional CSF1R expression, we investigated the ability of MPS cells to take up labeled pig CSF1-Fc fusion protein, which produces a large increase in tissue macrophage populations when injected into mice (12) or pigs (64). CSF1-Fc conjugated with AF647 (CSF1-Fc^AF647^) was found to bind specifically to monocytes in vitro (65). CSF1-Fc^AF647^ was injected intravenously 10 min before mice were sacrificed. In the liver, the uptake of CSF1-Fc^AF647^ was detected in KC, monocytes, and cDC2, but not in cDC1, pDC (Fig. 6A, 6B), or neutrophils (data not shown). Within cDC2, CSF1-Fc^AF647^ binding was prevalent in CD11c^hi^ cells (Fig. 6C) precluding any possible confusion with the CD11c^dim^ MHCII^+^ subcapsular macrophages described recently (42). In the lung, the majority of Ly6C^+^ monocytes and interstitial macrophages bound CSF1-Fc^AF647^, whereas both cDC populations were negative (Fig. 6A, 6B). Uptake of labeled CSF1-Fc by myeloid populations was reduced or abolished by the anti-CSF1R Ab, AFS98 (Fig. 6A, 6B), a weak inhibitor of receptor-ligand binding (66), and an identical labeling profile was observed following injection of a non–Fc-fused AF647-labeled porcine CSF1 (CSF1^AF647^) (data not shown). No detectable CSF1-Fc^AF647^ was bound by alveolar macrophages (Fig. 6A, 6B) and these cells also failed to bind appreciable levels in vitro (data not shown). Labeled anti-CD45 Ab was able to access all other myeloid populations in lung (data not shown) (67) and liver (Fig. 6D), suggesting a lack of CSF1-Fc^AF647^ or CSF1^AF647^ uptake by certain cDC reflects an absence of surface CSF1R expression rather than the inaccessibility of the sites they may occupy (31, 53).

Consistent with previous population-level data on CSF1 clearance (68), KC bound the highest level of labeled CSF1 per cell in a receptor-dependent manner (Fig. 6B), and considerably more per cell than blood monocytes (Fig. 6E). In turn, Ly6C^+^ monocytes in lung and liver were more intensely labeled than interstitial lung macrophages or liver CD11b^+^ cDC2 and liver Ly6C^−^ monocytes (Fig. 6B), although distinct intravascular versus parenchymal locations of these cells (31, 53) means exposure to circulating CSF1 cannot be controlled in this comparison. Of note, Ly6C^+^ blood monocytes, identified independently of CSF1R expression (Supplemental Fig. 1A), were more intensely labeled than the Ly6C^−^ subset (Fig. 6E), despite equivalent surface expression of CSF1R (Fig. 6F) (69, 70). Thus, novel differences in capacity to bind CSF1 were revealed using this ligand-binding approach.

**FIGURE 7.**
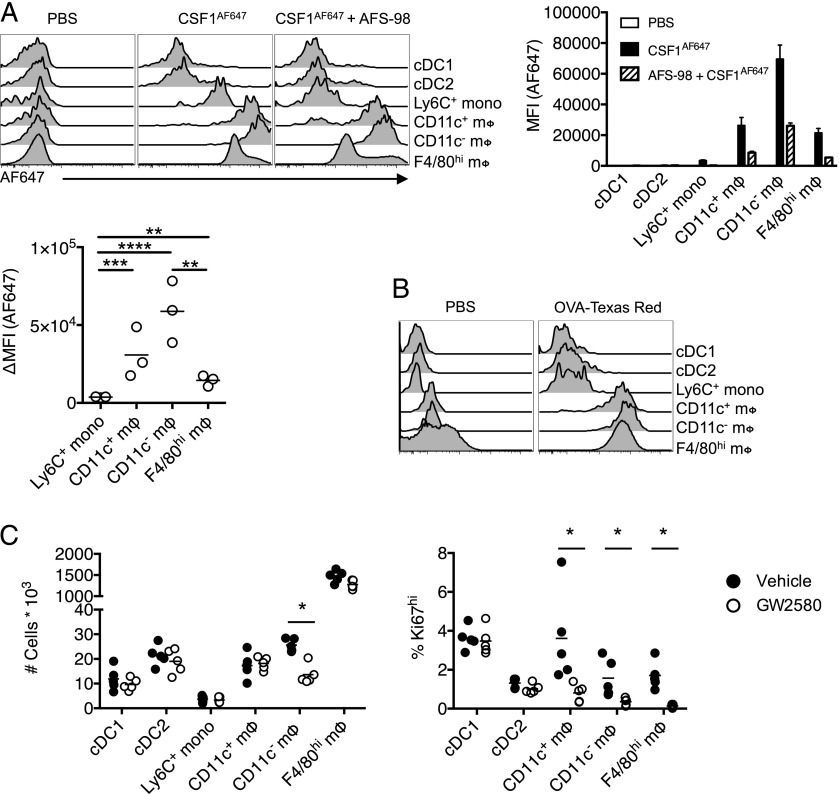
Consumption of CSF1 differs between monocytes and resident peritoneal macrophage populations. (**A**) Representative histograms of AF647 fluorescence in cavity leukocytes 10 min after i.p. injection of CSF1^AF647^ or PBS vehicle alone or in combination with pretreatment with AFS98, and graphs showing MFI of AF647 (right of histogram) and change in MFI between mice given CSF1^AF647^ alone or after AFS98 pretreatment (below histogram), with data presented as mean ± SEM of three to four mice per group. Data are from one of three independent repeat experiments. (**B**) Representative histograms of Texas Red fluorescence in cavity leukocytes 10 min after i.p. injection of OVA–Texas Red conjugate from a single experiment. (**C**) Total number of cavity leukocytes and proportion expressing high levels of Ki67 from mice treated for 4 d with GW2580 (open) or vehicle control (closed), with data points depicting individual mice. Data representative of two independent experiments. The asterisk (*) indicates significant differences using multiple *t* tests corrected for multiple comparisons using the Holm–Sidak method.

The relationship between *Csf1r*-mApple activity and CSF1R-mediated ligand uptake was also examined in the peritoneal cavity following i.p. injection. Monocytes and CD11c^+^ and CD11c^−^F4/80^lo^CD226^+^ macrophages (49, 50) were identified as described in Supplemental Fig. 1B, avoiding the use of Abs to CSF1R. Neither *Csf1r*-mApple^lo^ cDC population bound appreciable levels of CSF1^AF647^, whereas all three macrophage populations had higher levels of receptor-dependent uptake of CSF1^AF647^ than Ly6C^+^ cavity monocytes (Fig. 7A). Surprisingly, the CD11c^−^F4/80^lo^ macrophages exhibited the greatest uptake. These differences were not explained by differential levels of receptor-independent macropinocytosis as uptake of injected OVA–Texas Red was largely equivalent between populations (Fig. 7B). Anti-CSF1R mAb inhibited CSF1 uptake to a similar degree in each population, with a reduction between 63 and 75%. The difference in receptor activity appeared to have functional significance, as treatment of mice daily for 4 d with a CSF1R kinase inhibitor, GW2580 (71), which has been shown to inhibit proliferation of microglia (41) and pleural macrophages (11), partly depleted the CD11c^−^ subset of F4/80^lo^MHCII^+^ peritoneal macrophages alone (Fig. 7C, left graph), despite inhibiting proliferation (as evidenced by Ki67 staining) of all macrophage populations (Fig. 7C, right graph).

### Csf1r-mApple:ΔCsf1r-ECFP mice allow in situ imaging of distinct mononuclear phagocytes

**FIGURE 8.**
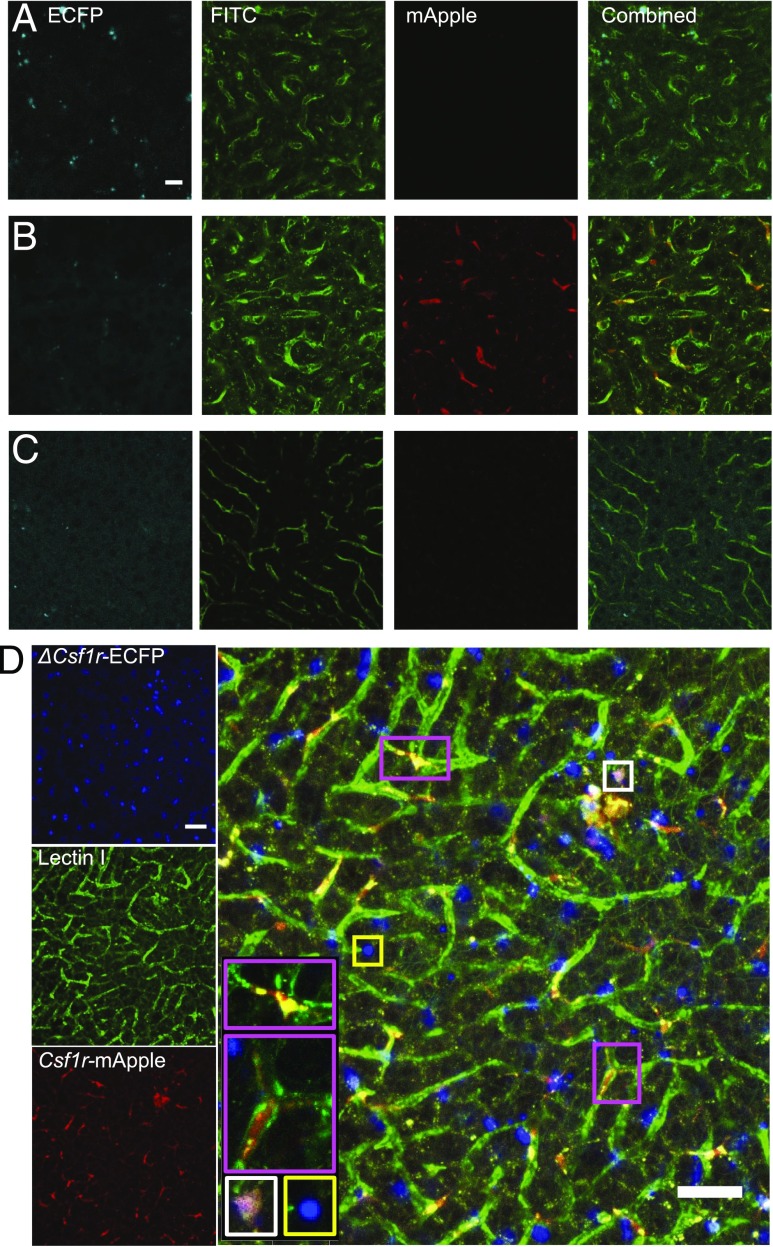
*Csf1r*-mApple and Δ*Csf1r*-ECFP transgenes allow imaging of distinct lineages of hepatic myeloid cells. Confocal image of the surface of the left lobe of the liver of a Δ*Csf1r*-ECFP (**A**), *Csf1r*-mApple (**B**), WT (**C**), and *Csf1r-*mApple/Δ*Csf1r*-ECFP (**D**) mouse imaged ex vivo. FITC-Lectin I was injected i.v. to reveal liver sinusoidal endothelium. Scale bars represent 20 μm (A–C) or 50 μm (D).

The Δ*Csf1r*-ECFP transgene was crossed previously to the *Cx3cr1^+/^*^gfp^ or *Itgax* (CD11c)-EYFP mouse to distinguish pulmonary monocytes from other myeloid lung populations (31). To determine the utility of the *Csf1r-*mApple mouse to facilitate in vivo imaging of different myeloid populations, we crossed it to the Δ*Csf1r*-ECFP line (32, 36). As in the intestine (32), the majority of cDC1, cDC2, and Ly6C^+^ and Ly6C^−^ monocytes in the liver expressed high levels of ECFP, whereas pDC expressed intermediate levels. Neutrophils, eosinophils, and lymphocytes were negative (data not shown) as were F4/80^hi^ KC (Supplemental Fig. 2A). All ECFP^+^ cells expressed intermediate levels of mApple (Supplemental Fig. 2B, cyan gate), whereas all mApple^hi^ cells were ECFP^−^ and represent KC (Supplemental Fig. 2B, red gate). We combined the two *Csf1r* reporters with detection of endothelial cells by injection of FITC-labeled Lectin I. In confocal images the mApple^+^ cells were almost completely restricted to the liver sinusoids (e.g., pink boxes), consistent with KC (Fig. 8). In contrast, ECFP^+^mApple^+^ double-positive cells were rarely detected (e.g., white box), despite the presence of numerous ECFP^+^ mApple^−^ cells (e.g., yellow box) (Fig. 8). These data suggest the intermediate levels of *Csf1r*-mApple expressed in ECFP^+^ cDC and monocytes (Fig. 5B, Supplemental Fig. 2A) are apparently below the threshold of detection of confocal imaging. This conclusion was supported by only weak detection of mApple expression when peripheral blood was imaged using identical microscope settings (Supplemental Fig. 3A). The *Csf1r*-ECFP^+^ cells in the liver (e.g., yellow box) were mainly detected outside the sinusoids and likely include the subcapsular liver macrophages that also express the Δ*Csf1r*-ECFP transgene (42). The high level of mApple expression in KC therefore allows imaging of these cells without detection of monocytes and other mApple^+^ cells in the liver.

**FIGURE 9.**
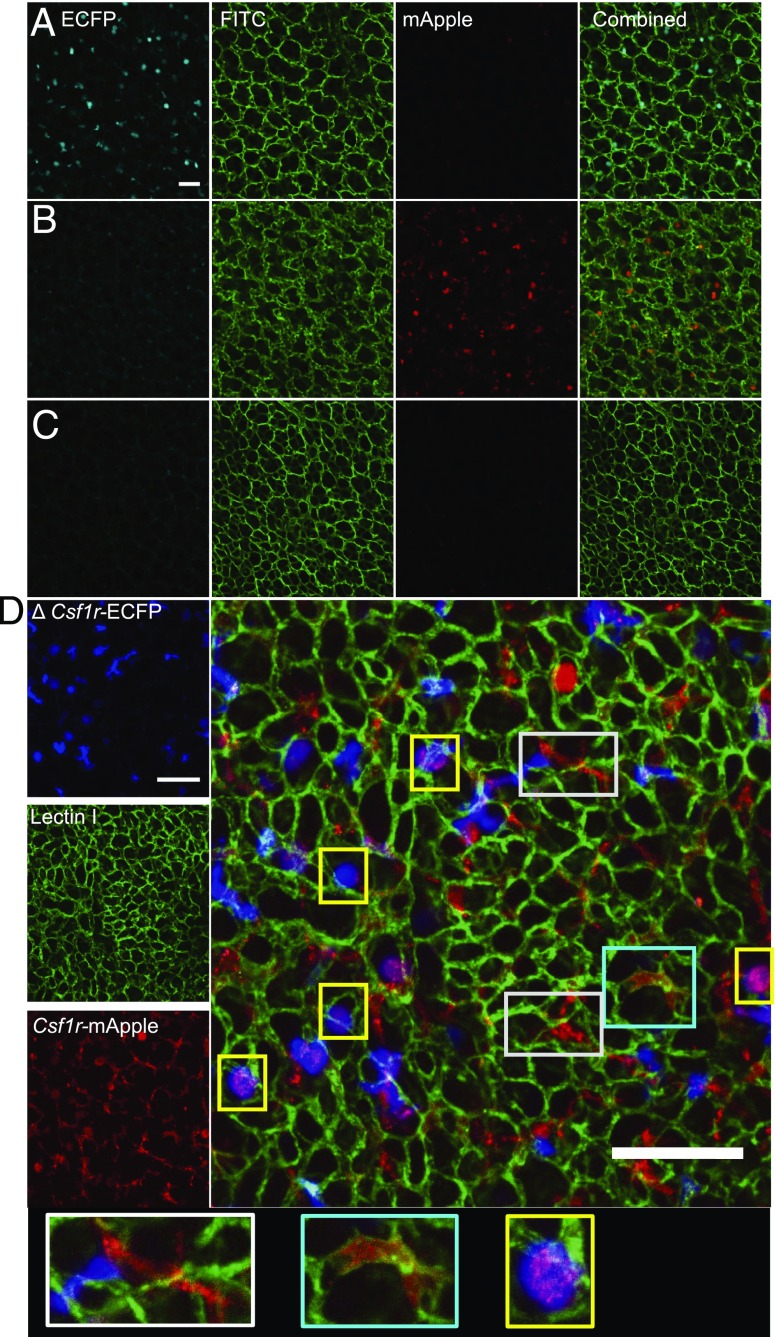
*Csf1r-*mApple and Δ*Csf1r*-ECFP transgenes allow imaging of distinct lineages of pulmonary myeloid cells. Confocal image of a transverse section of lung from a Δ*Csf1r*-ECFP (**A**), *Csf1r*-mApple (**B**), WT (**C**), and *Csf1r*-mApple/Δ*Csf1r*-ECFP (**D**) mouse imaged ex vivo. FITC-Lectin was injected i.v. to reveal pulmonary vasculature. Scale bars in all panels represent 50 μm.

In the lung, interstitial macrophages and DC were ECFP negative (Supplemental Fig. 2C) whereas the majority of alveolar macrophages and Ly6C^+^ monocytes expressed ECFP, as reported previously (31, 32). Combined, all ECFP^+^ cells were mApple^+^ (Supplemental Fig. 2D, cyan gate) and encompassed alveolar macrophages and Ly6C^+^ and Ly6C^−^ monocytes, whereas ECFP^−^mApple^hi^ cells (Supplemetal Fig. 2D, red gate) comprised CD64^+^ interstitial macrophages and a minor fraction of ECFP^−^ alveolar macrophages. In confocal images of transverse lung sections, parenchymal populations broadly divided into rounded ECFP^+^mApple^+^ cells (Fig. 9, yellow box) consistent with alveolar macrophages or interstitial migratory monocytes (31, 67) and elongated stellar-shaped ECFP^−^mApple^+^ cells (Fig. 9, white box). Injection of CSF1-Fc^AF647^ into the double transgenic mice selectively labeled the extravascular interstitial ECFP^−^mApple^+^ cells (Supplemental Fig. 4, white box), visible as punctate staining indicative of internalization of labeled ligand, and confirmed them to be interstitial macrophages rather than ECFP^−^mApple^lo^ pulmonary DC. Many extravascular ECFP^+^mApple^+^ cells also took up CSF1-Fc^AF647^ (Supplemental Fig. 4), most likely migratory monocytes identified previously by live imaging (30, 31). In contrast, the most frequent cells observed within the pulmonary capillaries were ECFP^−^mApple^+^ (Fig. 9D, cyan box) and failed to label with injected CSF1-Fc^AF647^ (Supplemental Fig. 4), consistent with pulmonary neutrophils (72). Consistent with this, imaging of blood cells at identical power settings confirmed strong detection of mApple in ECFP^+^ monocytes and ECFP^−^ neutrophils (Supplemental Fig. 3B).

### Heterogeneous expression of Csf1r reporter genes in the brain

**FIGURE 10.**
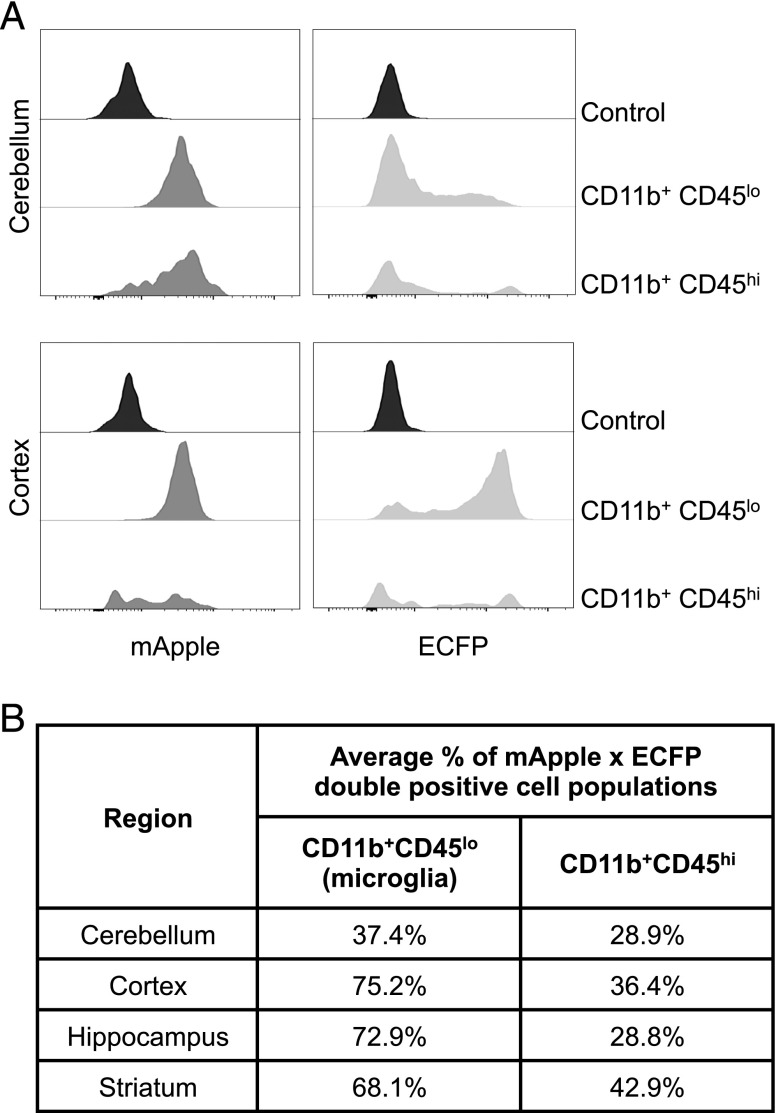
The expression of *Csf1r*-mApple and Δ*Csf1r*-ECFP transgenes in regional brain homogenates. Cerebellum, cortex, hippocampus, and striatum were processed to generate a single-cell suspension. (**A**) Compared are CD11b^+^CD45^lo^ microglia and CD11b^+^CD45^hi^ cells of the cerebellum and cortex regarding their expression of *Csf1r*-mApple and ECFP. (**B**) Percentage of double transgene positive CD11b^+^CD45^lo^ and CD11b^+^CD45^hi^ cell populations across all selected regions.

Macrophages in the mouse embryo are ECFP positive in the Δ*Csf1r*-ECFP line from their earliest appearance in the yolk sac (32). In addition to alveolar macrophages, one of the few locations in adults in which transgene expression is retained is in microglia. Grabert et al. (73) reported differences in microglia numbers and gene expression profiles in different mouse brain regions, and changes in gene expression with age. We used the *Csf1r*-mApple: Δ*Csf1r*-ECFP cross to further dissect microglial heterogeneity in different brain regions. CD45^lo^CD11b^+^ classical microglia were uniformly strongly positive for mApple (Fig. 10A). Like blood monocytes and alveolar macrophages, microglia in cortex, hippocampus, and striatum were largely positive for ECFP, but in the cerebellum the percentage was much lower (37.4%, Fig. 10B). The level of ECFP in these cells was also lower, and lacked a clear peak, reminiscent of the ECFP profiles of macrophages and DC in the gut (32). The brain also contains a separable CD11b^+^, CD45^hi^ macrophage-like microglial population, a subset of which occupies perivascular locations and expresses higher levels of *Csf1r* than monocytes (74). By contrast to the classical microglia and blood monocytes, the CD11b^+^CD45^hi^ cells were >50% ECFP negative in all brain regions (Fig. 10B).

## Discussion

We have developed a novel *Csf1r-*mApple reporter line. The *Csf1r* promoter construct used has been remarkably consistent in generating location and copy-number–independent expression of transgenes (75), further confirmed by the comparable pattern of *Csf1r*-mApple and *Csf1r-*EGFP transgene expression. Expression of *Csf1r*-mApple had no impact on numbers of tissue macrophages or circulating blood leukocytes (data not shown). With optimal microscope settings, the distinct profile of transgene expression across subsets of MPS cells allowed exclusive detection of mApple^hi^ cells. When combined with the Δ*Csf1r*-ECFP reporter gene, which selectively labels subsets of *Csf1r*-positive cells, CSF1-Fc labeled with AF647, and FITC-labeled Lectin, we could identify and image lung interstitial macrophages and liver KC, and distinguish them from other myeloid cells. Despite high levels of *Csf1r-*mApple and *Csf1r*-EGFP (46) transgene expression, neutrophils are identifiable by injection of labeled Abs to Ly6G, a molecule with a negligible role in neutrophil trafficking or function (76, 77). Thus, there are numerous possibilities to produce live images of macrophage behavior and heterogeneity, particularly by combining with other established EGFP-based reporter mice.

In common with the *Csf1r*-EGFP reporter (78), *Csf1r*-mApple expression was uniformly higher in resident macrophages compared with monocytes, a difference reflected in the ability of at least liver and cavity macrophages to capture more CSF1 on a per-cell basis than monocytes in vivo. Consistently, peritoneal macrophages compete effectively for available CSF1 in mixed culture with proliferating BM-derived macrophages (79). The rapid uptake of CSF1^AF647^ by KC is consistent with their role in regulating the circulating CSF1 concentration (68). Hence, upregulation of CSF1R expression may be a general feature of macrophage differentiation that allows them to compete for or control bioavailable CSF1. The apparent inability of alveolar macrophages to capture CSF1 is a notable departure from this tenet. However, alveolar macrophages are unaffected in adult CSF1-deficient op/op mice (80) and their replenishment from BM following irradiation is largely independent of CSF1R (81). Thus, our data are consistent with a lack of role for CSF1 in maintenance of the alveolar macrophage niche. However, it remains unclear why both alveolar macrophages and granulocytes express high levels of *Csf1r* transgene despite lacking a surface receptor. In the blood, Ly6C^+^ monocytes more readily took up CSF1 than their Ly6C^−^ progeny, a feature consistent with the suggestion that Ly6C^+^ monocytes regulate the availability of CSF1, thereby controlling the lifespan of the Ly6C^−^ population (57). Interestingly, higher consumption of CSF1 by classical monocytes is also evident in PBMCs from *Csf1r*-EGFP transgenic sheep (65), suggesting this feature is conserved across species.

Intensity of fluorescence in *Csf1r*-mApple mice also largely distinguished long-lived tissue-resident macrophages (KC, alveolar macrophages, and F4/80^hi^ peritoneal macrophages) from those of more recent monocyte-origin (F4/80^lo^ resident peritoneal and lung interstitial macrophages) but was not correlated with the ability to take up labeled CSF1. In the peritoneal cavity, the receptor activity was greatest in F4/80^lo^CD11c^−^ cells. Notably, these cells were selectively depleted following treatment with the CSF1R kinase inhibitor GW2580. Dynamics of loss of labeled histone 2B-GFP from peritoneal F4/80^lo^ macrophages places the half-life for replenishment of both CD11c^+^ and CD11c^−^ subsets from monocytes at around 2 wk (23), much longer than the 4 d treatment regimen in this study. Hence, selective loss of F4/80^lo^CD11c^−^ cells likely results from reduced survival or retention in the cavity rather than a failure of monocytes to differentiate and replenish these cells. Both F4/80^hi^ and F4/80^lo^ populations of peritoneal macrophages are rapidly lost upon Ab-mediated neutralization of CSF1 (24), suggesting only partial blockade of CSF1R signaling occurred with the oral inhibitor used in this study. Higher levels of CSF1R signaling are generally required for proliferation than survival of macrophages (8) and hence the uniform inhibition of Ki67 expression observed across the peritoneal macrophage compartment is consistent with a reduction of high-level CSF1R-signaling by GW2580 treatment. In light of this, our data suggest the F4/80^lo^CD11c^−^ subset require a higher threshold of CSF1R signaling for survival and consequently exhibit greater CSF1R activity. Thus, different populations of peritoneal macrophages would appear to pursue distinct survival strategies. Short-lived cells are more reliant on high levels of CSF1 signaling, whereas long-lived cells are better adapted to efficiently use CSF1, possibly explaining the predominance of the latter under homeostatic CSF1-limited conditions (82). Either way, our data reveal fine-tuning of CSF1R activity but not necessarily *Csf1r* transgene or gene expression between distinct macrophage populations.

Relatively low, or absent, expression of the *Csf1r*-mApple reporter also provided a useful marker delineating peritoneal and pulmonary CCR2-independent cDC from CCR2-dependent CD11c^+/−^MHCII^+^ APC, likely of monocyte origin. Monocyte-derived CD11c^+^ APC have also been described within the dermis, kidney, and gut (6, 21, 54, 83), in which tissue cDC also expressed lower levels of *Csf1r* (22). In the liver, MHCII^+^CD11c^+^ cells were largely replenished by CCR2-independent BM precursors, uniformly expressed the candidate cDC marker CD26, and lacked the candidate macrophage marker CD64. Consistent with a cDC nature, these cells also uniformly express the transcription factor Zbtb46 (84). Nevertheless, these cells showed similar levels of *Csf1r-*mApple transgene expression to monocytes, and the CD11b^+^ cDC2 fraction bound labeled CSF1-Fc. Although juvenile *Csf1r*^−/−^ mice have normal numbers of hepatic cDC2 (14), unlike in other tissues, these cells also do not require CSF2 for survival (85), indicating possible redundancy between these growth factors. In adult mice the impact of *Csf1* and *Csf1r* mutations are more apparent (86) and anti-CSF1R treatment produced an almost complete depletion of liver cells expressing a *Csf1r*-EGFP transgene (87). Hence, in general, our data do not support an absolute division between Csf1r and Flt3-dependent APC populations. By analogy with the functional diversity of classical macrophages in different organs (51, 88–90), APC differentiation is likely also organ specific. Because CSF1 drives a largely immunoregulatory program (91), the responsiveness of cDC2 to CSF1 may underlie the relatively weak APC activity in liver (92) and contribute to a tolerogenic environment in the liver (93). Similarly, competition of CSF1R^+^ cDC2 together with KC and classical patrolling monocytes (94) for available CSF1 could provide an explanation for the relative absence of hepatic monocyte-derived MHCII^+^ APC.

In adult mice, labeling of cDC and macrophages in the Δ*Csf1r*-ECFP reporter is tissue specific (32). Using the Δ*Csf1r*-ECFP transgene, we highlighted the utility of the *Csf1r*-mApple strain to be crossed to existing reporter lines, visualizing distinct MPS populations in the lung and liver. Moreover, combined analysis of the *Csf1r*-mApple and Δ*Csf1r*-ECFP transgenes highlighted heterogeneity among microglia. Intriguingly, the percentages of Δ*Csf1r*-ECFP negative microglia correlated with the retention of microglia in the IL-34–knockout mouse in the same brain regions (95). Similarly, in other tissues, ECFP expression occurs predominantly in locations where macrophages are more reliant on IL-34 (for example, Langerhans cells) or CSF2 (alveolar macrophages). Hence, the graded expression of the Δ*Csf1r*-ECFP transgene in microglia may reflect its induction during differentiation or the proximity of individual cells to the tissue-specific factors that control its expression.

In overview, the *Csf1r-*mApple mouse recapitulates the expression profile of the widely used *Csf1r*-EGFP reporter. In combination with other reporters, and labeled CSF1, the *Csf1r-*mApple mouse provides a new tool to dissect the differentiation and function of the heterogeneous populations of mouse tissue mononuclear phagocytes and the homeostatic roles of CSF1. How different mononuclear phagocytes regulate CSF1R activity remains an important question given the continued interest in macrophages as possible vehicles for delivery of gene therapies and as targets of therapeutics.

## Supplementary Material

Data Supplement
