# Supplementary material for: Csf1r-mApple Transgene Expression and Ligand Binding In Vivo Reveal Dynamics of CSF1R Expression within the Mononuclear Phagocyte System
Source: J Immunol. 2018 Feb 12;200(6):2209–23. doi: 10.4049/jimmunol.1701488 (PMC5834790; doi:10.4049/jimmunol.1701488)
Supplement: Data Supplement [file JI_1701488.zip › JI_1701488_Supplemental_Figures_1.pdf]

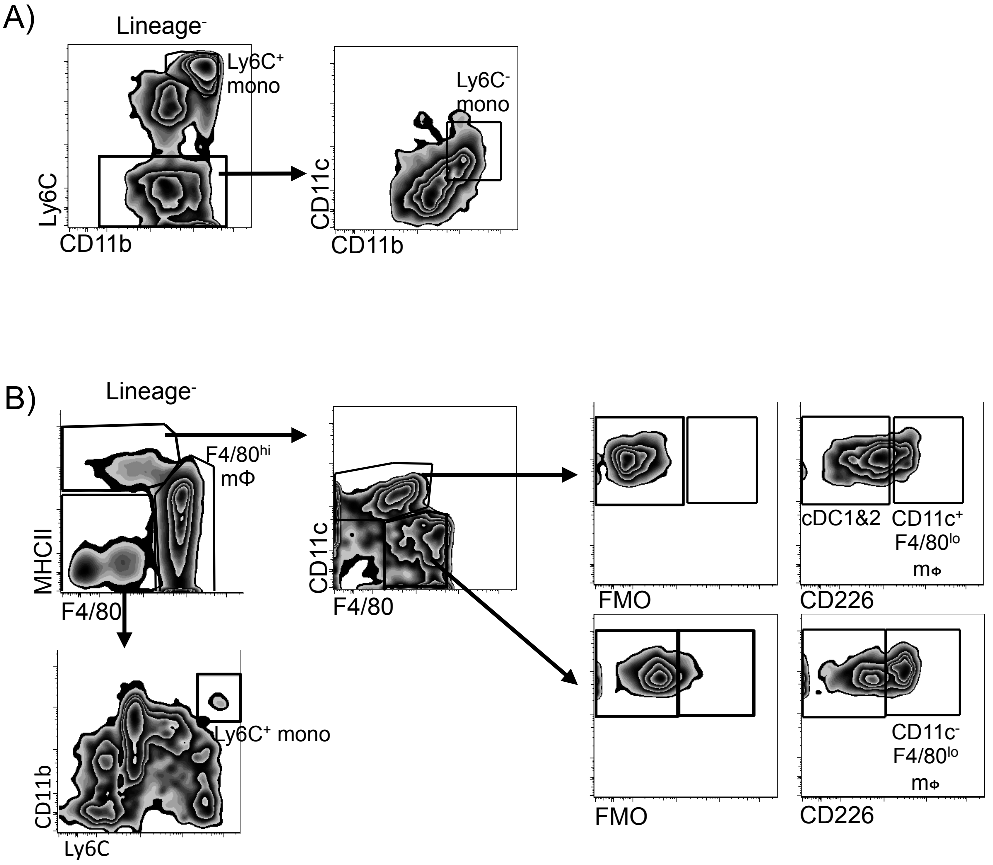

SFigure 1: Alternative gating strategies to identify blood monocytes and peritoneal myeloid populations without CSF1R expression. (A) $Ly6C^{+}$ and $Ly6C^{-}$ blood monocytes were identified by high levels of Ly6C and CD11b (left plot), or CD11c and CD11b (right plot), respectively. **(B)** $Ly6C^{+}$ cavity monocytes were identified as $MHCII^{-}CD11b^{hi}Ly6C^{hi}$, while CD226 was used to identify mature $CD11c^{-}MHCII^{+}F4/80^{lo}$ macrophages and distinguish $CD11c^{+}MHCII^{+}F4/80^{lo}$ macrophages from $CD11c^{+}F4/80^{lo}$ cDC. cDC were further split into $CD11b^{-}$ cDC1 and $CD11b^{+}$ cDC2 (not shown).

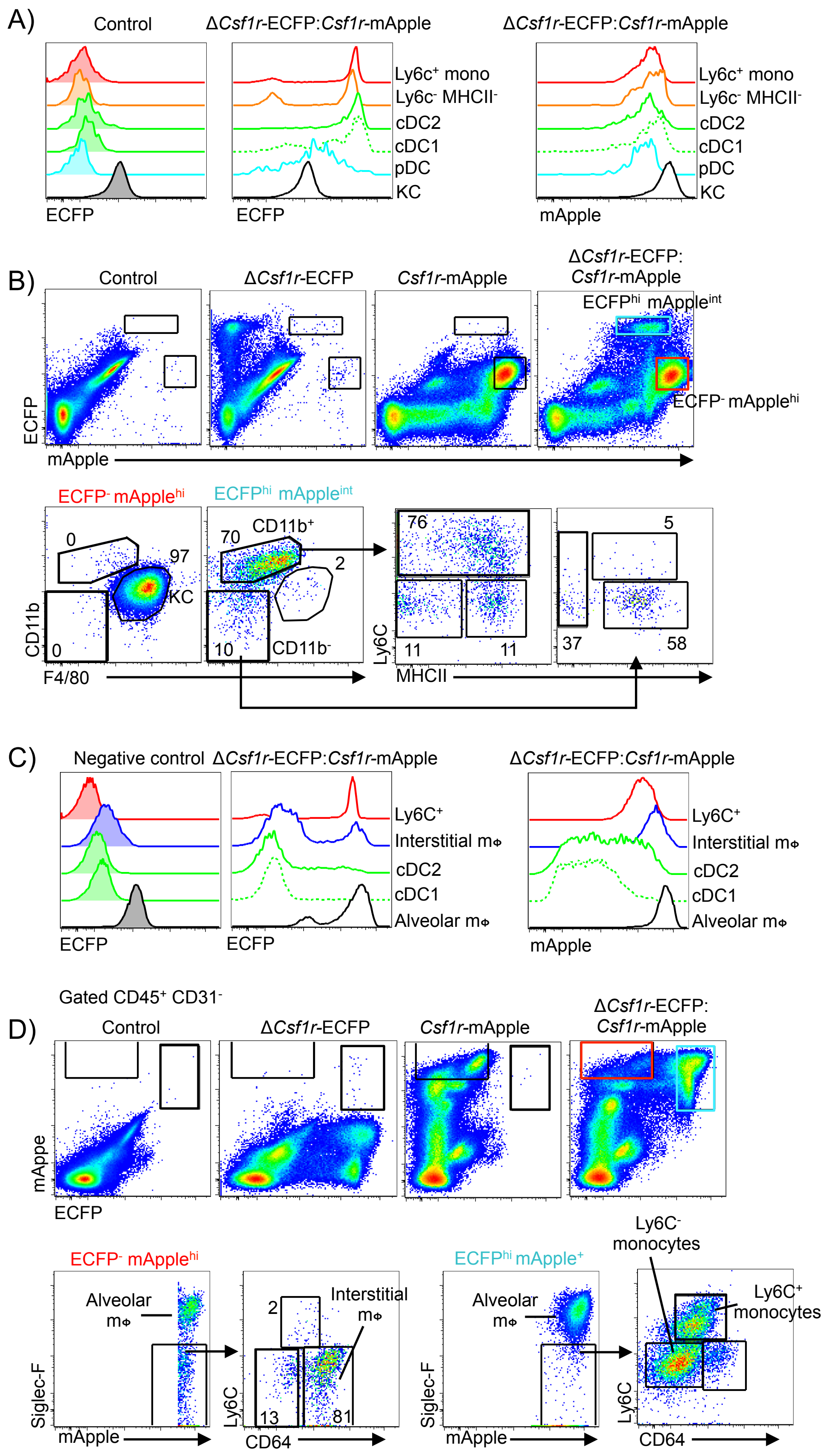

SFigure 2: (A) Representative ECFP and mApple expression by hepatic myeloid subsets from *Csf1r*-mApple:Δ*Csf1r*-ECFP double transgenic and control mouse. **(B)** Gates depicting all ECFP^{+} mApple^{+} cells (green) and mApple^{hi} ECFP^{-} cells (red) within CD45^{+} hepatic cells from a *Csf1r*-mApple:Δ*Csf1r*-ECFP mouse and single *Csf1r*-mApple or Δ*Csf1r*-ECFP or negative controls, and surface marker expression and frequencies of cells falling within these gates. Data is representative of 1-2 mice per group and from 1 representative experiment of 2. **(C)** As A but for pulmonary myeloid subsets **(D)** Gates depicting all ECFP^{+} mApple^{+} cells (blue) and mApple^{hi} ECFP^{-} cells (red) within CD45^{+} pulmonary cells from a *Csf1r*-mApple:Δ*Csf1r*-ECFP mouse and single *Csf1r*-mApple, Δ*Csf1r*-ECFP, or negative controls, and surface marker expression and frequencies of Ly6C^{+} CD64^{dim} monocytes, CD64^{+} Ly6C^{-} interstitial macrophages and Ly6C^{-} CD64^{-} monocytes falling within these gates after Siglec-F^{+} alveolar macrophages were first excluded. Data is representative of 1-2 mice per group and from 1 representative experiment of 2.

SFig. 3

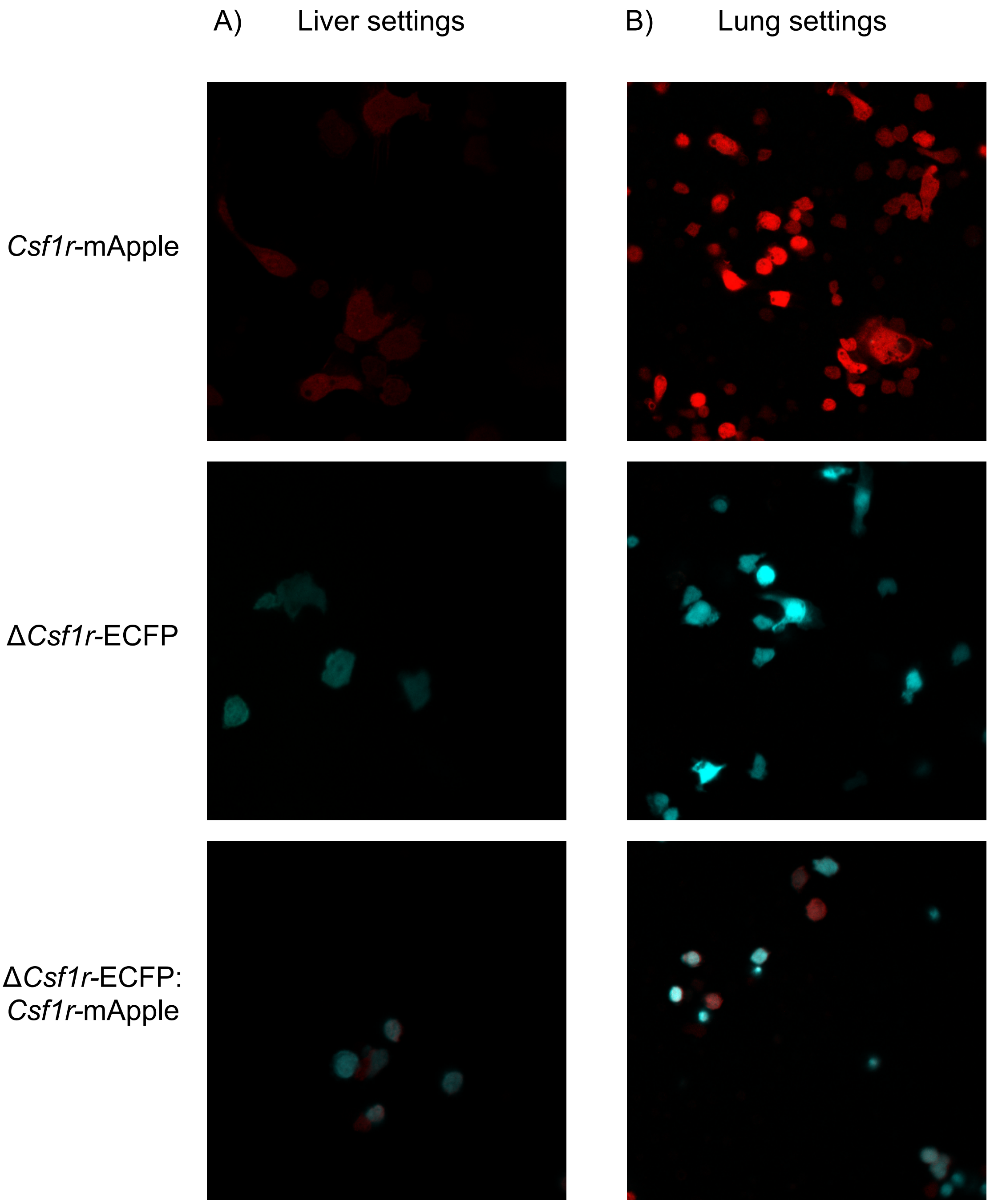

SFigure 3: Combined confocal images of mApple and ECFP in blood cells from *Csf1r*-mApple, Δ*Csf1r*-ECFP or *Csf1r*-mApple:Δ*Csf1r*-ECFP double transgenic mice using microscope settings used to image the liver **(A)** and lung **(B)** in Fig. 8 and Fig. 9 respectively.

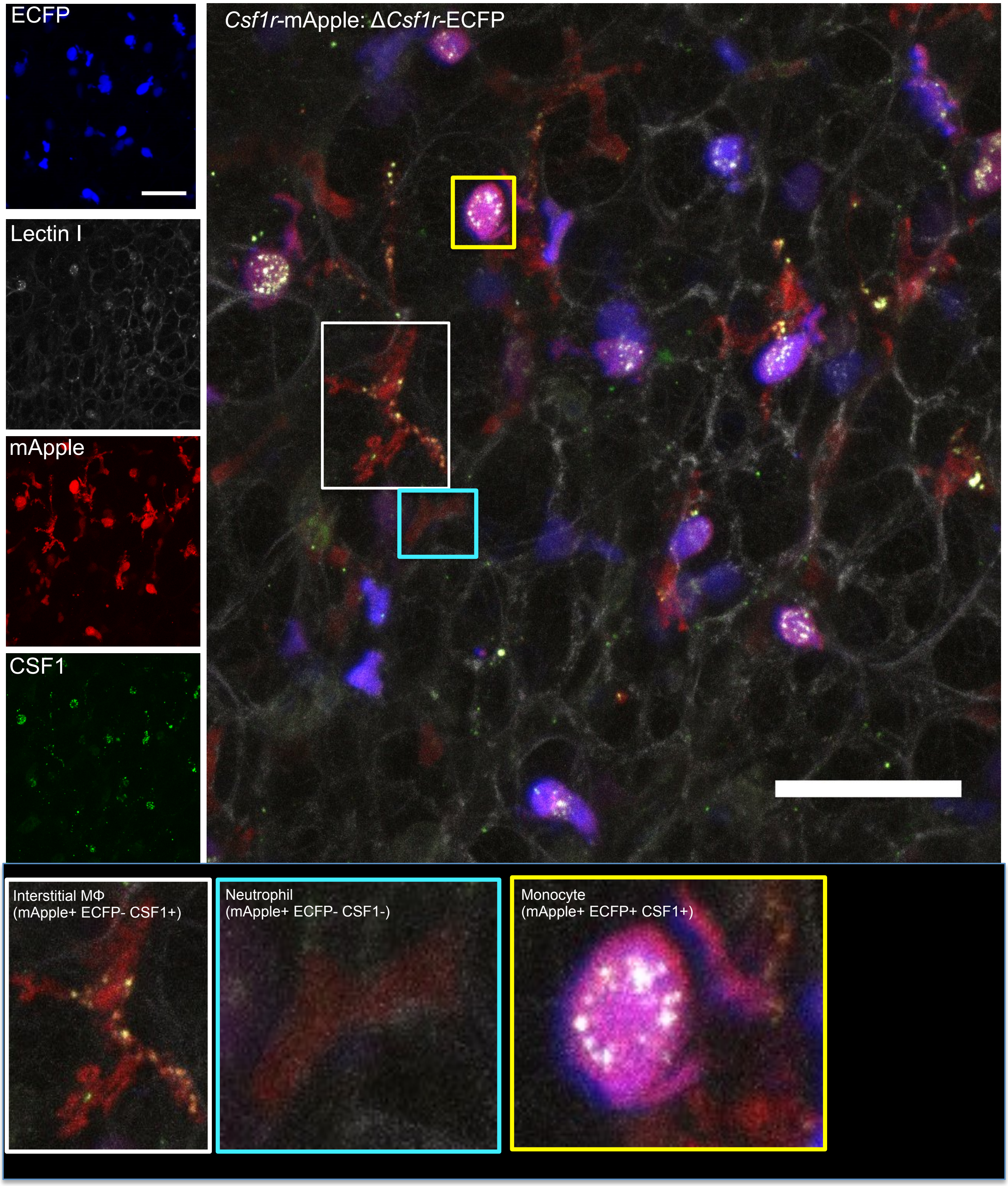

SFigure 4: CSF1^{AF647} labelling in *Csf1r*-mApple:Δ*Csf1r*-ECFP double transgenic mice confirms interstitial macrophage identity. Confocal image of a transverse section of lung from a *Csf1r*-mApple:Δ*Csf1r*-ECFP double transgenic mouse injected with FITC-Lectin I and CSF1^{AF647} i.v. to reveal pulmonary vasculature and CSF1R expression. Microscope acquisition settings match those from Fig. 9 using 8% laser power. Autofluorescence was detected using non-fluorescent tissue, and false-positive signal was discarded by using tissues that were single-positive for ECFP, mApple, FITC or AF647.
